# Multicomponent reactions driving the discovery and optimization of agents targeting central nervous system pathologies

**DOI:** 10.3762/bjoc.20.261

**Published:** 2024-12-03

**Authors:** Lucía Campos-Prieto, Aitor García-Rey, Eddy Sotelo, Ana Mallo-Abreu

**Affiliations:** 1 Centro Singular de Investigación en Química Biolóxica e Materiais Moleculares (CIQUS), Universidade de Santiago de Compostela, 15782 Santiago de Compostela, Spain; 2 Departamento de Química Orgánica, Facultade de Farmacia, Universidade de Santiago de Compostela, 15782 Santiago de Compostela, Spain; 3 Laboratory of Medicinal Chemistry (CSIC Associated Unit), Faculty of Pharmacy and Food Sciences, University of Barcelona, Av. Joan XXIII, 27-31, E-08028 Barcelona, Spain; 4 Institute of Biomedicine of the University of Barcelona (IBUB), Av. Diagonal 643, E-08028 Barcelona, Spain

**Keywords:** Alzheimer, depression, epilepsy, multicomponent reactions, Parkinson, schizophrenia

## Abstract

The ongoing quest to discover effective treatments for diseases remains a significant challenge for the scientific community. Multicomponent reactions (MCRs) have emerged as powerful tools in accelerating drug discovery, enabling the rapid generation of chemical libraries with high diversity in a time-efficient and environmentally sustainable manner. In this review, we focus on central nervous system (CNS) disorders, particularly Alzheimer's disease, Parkinson's disease, schizophrenia, depression, and epilepsy, where MCRs have contributed to the development of promising ligands in recent years. Rather than providing an exhaustive overview, this review aims to highlight key studies that address major CNS pathologies, relevant drug targets, and various MCR approaches. We have carefully selected representative articles and apologize to the authors whose important contributions may not be included. By concentrating on these pivotal studies, we strive to offer a clear and concise perspective on current research trends and breakthroughs in this field.

## Introduction

### Multicomponent reactions

Due to efforts to develop new drug arsenals faster to overcome the difficulties of medicinal chemistry (multistage and highly wasteful processes), multicomponent reactions (MCRs) have emerged as a plausible solution. MCRs are convergent reactions in which three or more reagents are combined in one step to yield a single product that contains most of the atoms from the starting materials [1–2]. Among the advantages of MCRs are their high atom economy and efficiency in bond formation, along with the cost-effectiveness of separating and purifying products [3]. These aspects are aligned with the goal of addressing concerns related to the generation of environmentally hazardous waste [4]. In addition, this type of reaction allows for the efficient discovery of low molecular weight compounds. As a result, most of these compounds are drug-like according to Lipinski's rule of five (Ro5) [5].

**Figure 1:**
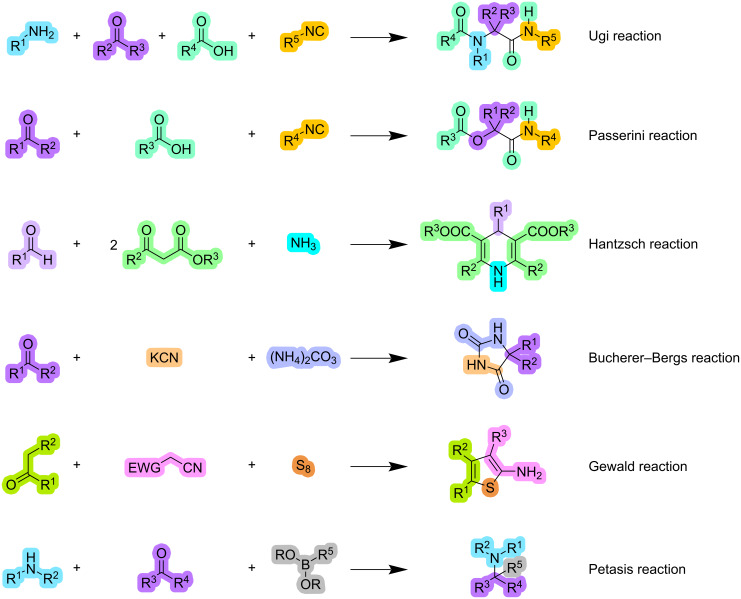
Classical MCRs.

Classical examples of MCRs include Ugi [6], Passerini [7], Hantzsch [8], Bucherer-Bergs [9], Gewald [10], and Petasis reactions [11], among others (Figure 1). Additionally, recent advancements have been seen in the discovery of novel MCRs by combination with other approaches such as visible light, microwaves, heterogeneous catalysis, and ultrasound [12–15].

Due to its versatility, one of the most prevalent of these MCRs is the Ugi reaction [16]. This reaction generally combines an isocyanide with an acid, an amine, and an aldehyde or ketone to obtain α-acylaminoamides. Innovation in recent years with alternative reagents, like *N*-hydroxyimides or nitric acid in place of an acid, *N*-alkylated hydrazines, or nitrobenzene derivatives (reduced in situ to anilines) instead of amines, and in situ-prepared isocyanides, makes it a versatile method for synthesizing diverse scaffolds [6].

**Figure 2:**
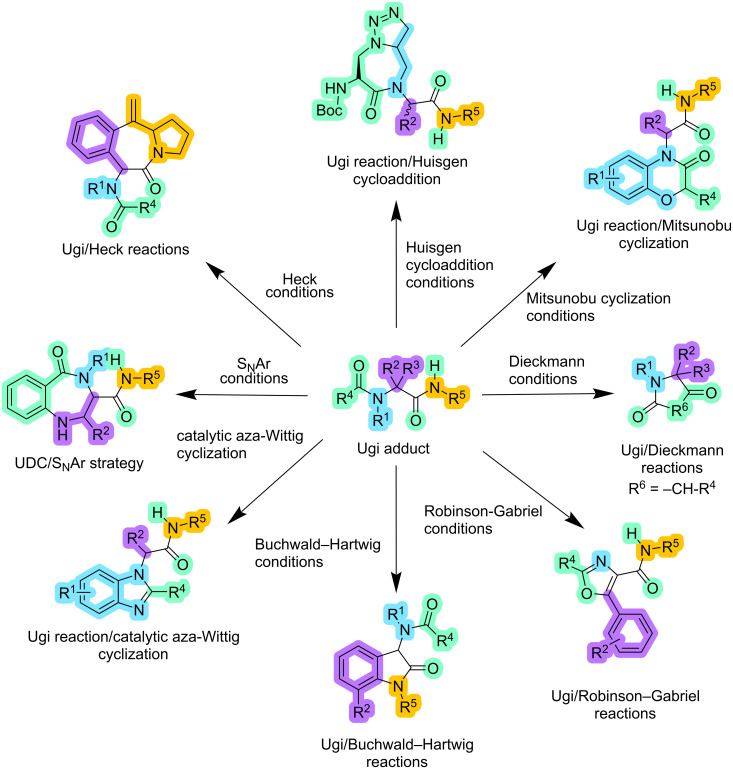
Different scaffolds that can be formed with the Ugi adduct.

Furthermore, the Ugi reaction exhibits versatility in forming both fused and unfused heterocyclic compounds, involving 4-, 5-, 6-, and 7-membered rings [17]. This capability is exploited by various approaches, including the Ugi–Deprotection–Cyclization strategy (UDC) and post-modification reactions. Examples of these reactions include the Ugi/Dieckmann reactions, Ugi/Robinson–Gabriel reactions, Ugi/Buchwald–Hartwig reactions, Ugi reaction followed by a catalytic aza-Wittig cyclization, Ugi reaction followed by SNAr strategy, Ugi/Heck reactions, Ugi/Huisgen cycloaddition, and Ugi reactions followed by Mitsunobu cyclization, among other strategies (Figure 2) [16].

### Central nervous system diseases

Central nervous system (CNS) diseases are the main cause of disability and the second cause of mortality worldwide, indeed, according to the 2019 World Health Organization report stroke ranked second among the leading causes of disease globally, followed by Alzheimer's disease (AD), and other dementias [18–19]. CNS diseases can be classified according to the etiological and pathological features into traumatic diseases, such as spinal cord and traumatic brain injuries, neurodegenerative diseases, such as AD and Parkinson’s disease (PD), demyelinating diseases such as multiple sclerosis (MS), etc. [20].

However, despite advances in the etiology of psychosis indicating both polygenetic and environmental factors, significant gaps remain [21]. Psychosis is a subgroup of severe mental illnesses characterized by delusions, hallucinations, and disorganized behavior. This illness can manifest itself across a spectrum of conditions such as major depressive disorder (MDD), bipolar disorder, delusional and schizophreniform disorder. The prototypical psychotic disorder is schizophrenia, which is a chronic and debilitating condition whose etiology is unclear and about which there are many different hypotheses [20–21]. In the case of MDD, even though the etiology is unknown, there are several models that have focused on genes, environmental factors, and gene–environment interactions [22]. In relation to epilepsy, the etiology of seizures depends on age. In children, seizures are frequently originated by genetic factors, malformations of cortical evolution, and injuries from perinatal events. In adults lacking an inherited tendency, typical causes include encephalitis or meningitis, traumatic brain injuries, and brain tumors. Finally, in elderhood, seizures are often linked to primary craniocephalic trauma, brain tumors, and neurodegenerative disorders [23].

This review centers on five neurological disorders where ligands obtained using multicomponent reactions (MCRs) have been identified: Alzheimer’s disease, Parkinson’s disease, depression, schizophrenia, and epilepsy. Our goal is not to provide a comprehensive survey but rather to spotlight key studies that address significant CNS disorders, pertinent drug targets, and various MCR methodologies. We have thoughtfully selected representative articles and extend our apologies to authors whose important works may not be included. By focusing on these studies, we aim to present a clear and concise view of current research directions and significant advancements in the field.

## Review

### Ligands targeting CNS diseases obtained from MCR approaches

#### Alzheimer’s disease (AD)

The pathogenesis of AD is still not clear but is marked as a multifactorial disease [24], including a good deal of hypotheses involving the cholinergic hypothesis, glutamate excitotoxicity, tau aggregation, abnormal extracellular accumulation of Aβ peptides, and oxidative stress (OS) [25].

Alzheimer’s disease (AD) is commonly thought to emerge from a deficiency of the neurotransmitter acetylcholine (ACh) in neuronal and neuromuscular regions, according to the most recognized theory. This deficit may be caused by reduced synthesis or enzymatic function of acetylcholinesterase (AChE) on ACh. Among the five drugs prescribed for AD, four (donepezil, rivastigmine, galantamine, and tacrine) are built upon the cholinergic hypothesis [26]. In contrast, memantine emerges as the only drug available that targets the glutamatergic system for AD, functioning as a non-competitive antagonist of the *N*-methyl-ᴅ-aspartate (NMDA) receptor to regulate excessive glutamate stimulation [27]. The β-amyloid hypothesis for AD is based on the formation of senile plaques, which are formed after the accumulation of insoluble aggregates of β-amyloid protein, primarily Aβ-42, in the brain. The gene for amyloid precursor protein (APP), found on chromosome 21, undergoes a mutation that causes the production of Aβ-42 instead of Aβ-40. APP, a transmembrane protein, is typically cleaved by α-secretase and γ-secretase, which leads to the formation of soluble Aβ-40. However, when cleaved by β-secretase and γ-secretase, it produces Aβ-42. This form misfolds, leading to the formation of insoluble protein aggregates known as senile plaques, causing toxicity [28–29]. Oxidative stress occurs when there is an imbalance between prooxidants and antioxidants in the body, leading to damage in various biomolecules like DNA and lipids. Lipid peroxidation and membrane disruption can cause random cross-linking, resulting in cell death and the fragmentation of proteins and enzymes. Elevated concentrations of reactive oxygen species (ROS) from various molecular processes contribute to the oxidation of proteins, nucleic acids, polysaccharides, and lipids. These modifications can impact the structure and biological functions of these molecules, ultimately leading to neuronal death, as observed in conditions like AD [30–31].

Recent studies highlight OS as an early event in AD pathogenesis, and mitochondria-directed and metabolic antioxidants have shown efficacy in AD mouse models, with ongoing trials for vitamin E and selenium [32].

**Scheme 1:**
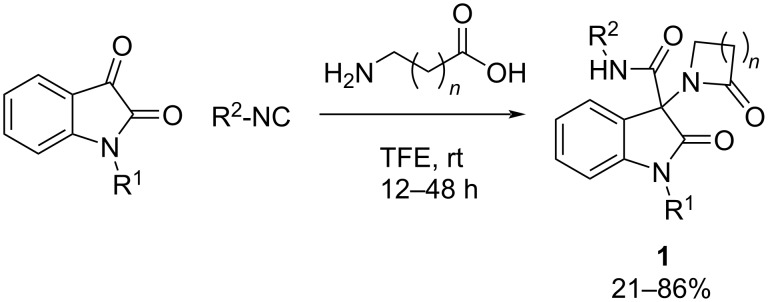
Oxoindole-β-lactam core produced in a U4C-3CR.

**Ugi reaction:** As already mentioned, one of the main targets in AD treatment are cholinesterase ligands. In recent years, butyrylcholinesterase, an enzyme present in high concentrations in the later stages of this pathology, has been gaining attention for the treatment of this disease. In 2021, Brandão et al. [33] developed a series of molecules inspired by the oxoindole-β-lactam core, a structural motif, present in many acetylcholinesterase inhibitor drugs, through the Ugi reaction (Scheme 1). A wide variety of isatins were suitable for performing the 4-center 3-component Ugi reaction (U4C-3CR), as well as isocyanides. Using 3-aminopropanoic acid (or β-alanine) and 4-aminobutanoic acid (or γ-aminobutyric acid), the corresponding β- and γ-lactam derivatives were obtained, respectively.

**Figure 3:**
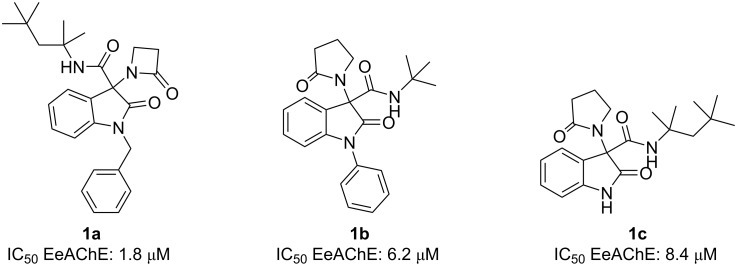
Most active oxoindole-β-lactam compounds developed by Brãndao et al. [33].

For the evaluation of the inhibitory activity of the compounds, model cholinesterases type VI-S, purified from *Electrophorus electricus* (EeAChE) and butyrylcholinesterase, purified from equine serum (eqBuChE) were employed. Only one compound showed moderate inhibitory activity (IC_50_ = 45 μM) on EeAChE. On the other hand, the inhibitory activity on eqBuChE was more promising, with the best scoring compounds being **1a**, **1b**, and **1c** (Figure 3). These compounds have mixed inhibitory activity (they can bind both the free enzyme and the enzyme–substrate complex, E–S complex). Additionally, compound **1a** showed more potency than galantamine (IC_50_ = 3.9 μM), a cholinesterase inhibitor in clinical use against AD.

**Scheme 2:**
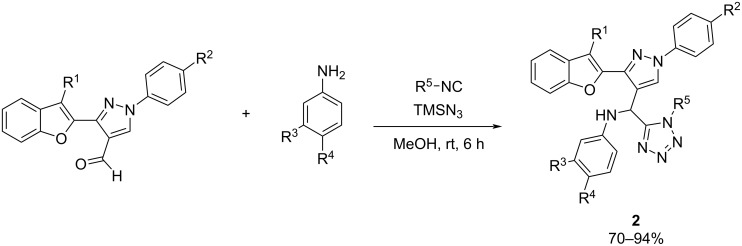
Ugi-azide synthesis of benzofuran, pyrazole and tetrazole hybrids.

In the search for novel ligands for Alzheimer's treatment, a common strategy involves the combination of structural fragments from different molecules. This concept often yields new compounds with improved biological activities. Employing this approach, Kushwaha et al. [34] synthesized a series of compounds containing benzofuran, tetrazole, and pyrazole within a single structure (Scheme 2). Benzofurans and tetrazole pharmacophores have demonstrated potential anti-Alzheimer activity by inhibiting AChE, while pyrazole scaffolds possess the ability to reduce the tau and β-amyloid dual aggregation. Benzofuran-pyrazole aldehydes were employed in the Ugi azide reaction to give the desired hybrids.

**Figure 4:**
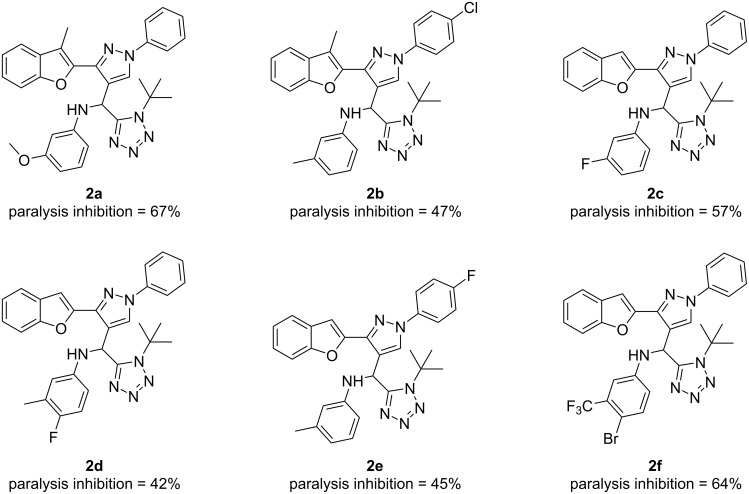
The most promising hybrids synthesized via the Ugi-azide multicomponent reaction reported by Kushwaha et al. [34].

From the screened compounds, **2a**, **2b**, **2c**, **2d**, **2e**, and **2f** demonstrated notable efficacy in regulating the paralysis rate of worms (Figure 4). Specifically, they exhibited a percentage of paralysis inhibition of 67%, 47%, 57%, 42%, 45%, and 64%, respectively, which indicates their potential in inhibiting β-amyloid toxicity in AD. These compounds were selected to assess their potential as acetylcholinesterase inhibitors, and their effects were investigated using the Amplex-Red^®^ kit in *C. elegans*. Individuals exposed to compounds **2a**, **2b**, **2c**, **2d**, **2e**, and **2f** at a concentration of 1 mM were analyzed for AChE activity in their extracts. The results revealed significant inhibition of AChE levels, with compound **2e** demonstrating the highest potential among the tested compounds against acetylcholinesterase.

**Scheme 3:**
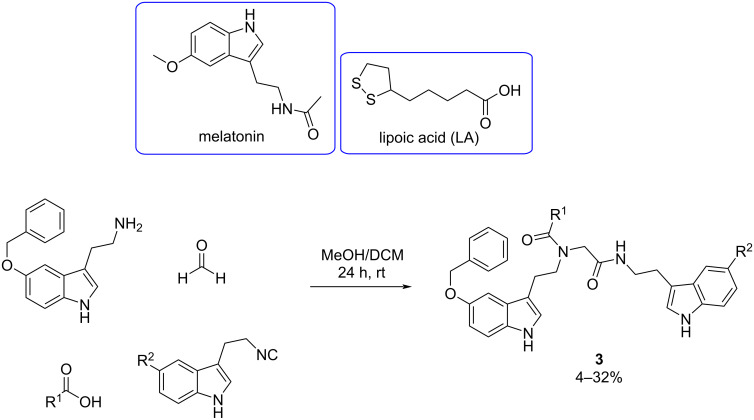
Four-component Ugi reaction for the synthesis of novel antioxidant compounds.

Furthermore, Pachón-Angona et al. [35] synthesized hybrid molecules using the Ugi reaction (Scheme 3), with antioxidant compounds as starting substrates, pursuing the aim of developing more powerful antioxidants.

The antioxidant properties were determined by oxygen radical absorbance capacity assay (ORAC-FL), expressed as Trolox equivalents (TE) and using fluorescein as probe. Additionally, the study investigated the ability of the compounds to activate the nuclear factor (erythroid-derived 2)-like 2 (Nrf2) pathway in cells containing the Nrf2/antioxidant response element (ARE).

**Figure 5:**
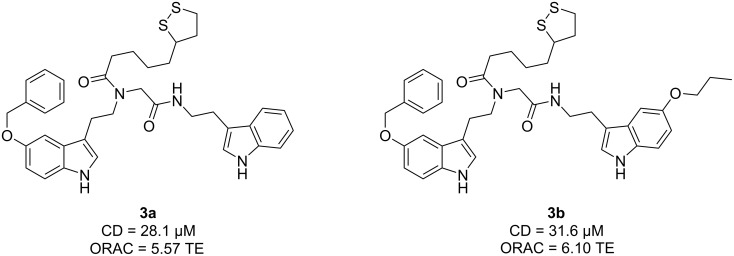
Most potent antioxidant compounds obtained through the Ugi four-component reaction developed by Pachón-Angona et al. [35].

The melatonin–lipoic acid (LA) hybrids **3a** and **3b** emerged as potent antioxidants, successfully inducing the Nrf2 transcriptional pathway at concentrations of 10, 30, and 100 μM (Figure 5). Notably, these hybrids exhibited lower CD values (28.1 and 31.6 μM, respectively), which is the concentration required to double the specific luciferase reporter activity, compared to melatonin (CD = 66.4 µM). The results suggest that compounds **3a** and **3b** are promising as potent antioxidant therapeutic agents. Furthermore, the results of the ORAC-FL assay were also remarkable, yielding a value 2-fold higher than melatonin (ORAC = 2.45 TE).

**Scheme 4:**
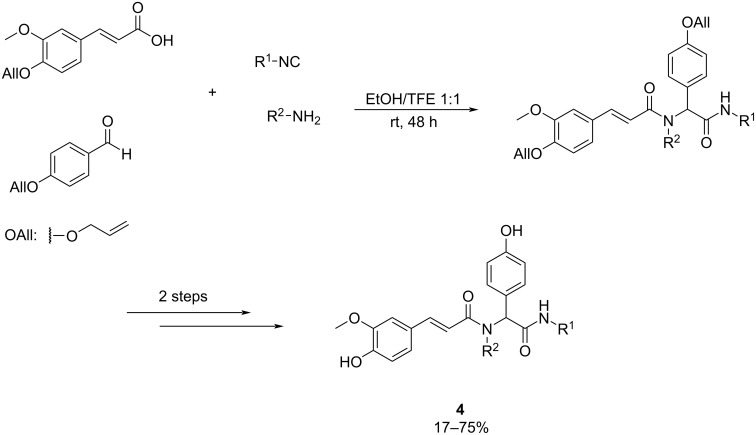
Four-component Ugi reaction to synthesize β-amiloyd aggregation inhibitors.

Natural polyphenols have shown promising potential in inhibiting β-amyloid aggregation and also antioxidant properties, suggesting their effectiveness in preventing AD. To address this, Lambruschini et al. [36] developed artificial, ‘natural-like’ polyphenols, using the Ugi reaction, since it leads to mixed polyphenol–peptidomimetic structures (Scheme 4).

The procedure involved an Ugi reaction using phenolic building blocks protected as allyl ethers, followed by deprotection, acetylation, and high-yielding solvolysis of the polyacetates. Also, the Ugi reaction gave the best results when imine preformation was implemented. In this preliminary paper, it was observed that the ferulic derived part was highly significant and for this reason, further experiments were conducted by Galante et al. [37] maintaining the carboxylic building block.

**Figure 6:**
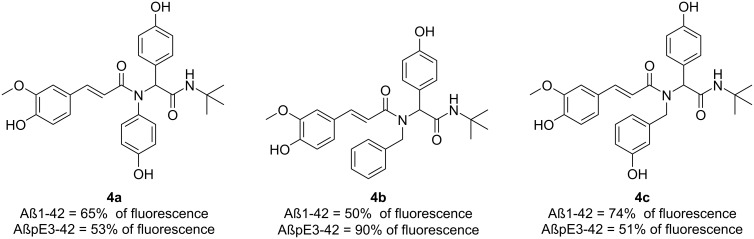
The most potential β-amiloyd aggregation inhibitors generated by Galante et al. [37].

The obtained molecules were evaluated for their ability to inhibit β-amyloid aggregation by the interaction with two β‐amyloid peptides, Aβ1‐42 and AβpE3‐42. The aggregation inhibition experiment, which measures the decrease of fluorescence, was carried out with the thioflavin T methodology highlighting compounds **4a** (Aβ1‐42 = 65% and AβpE3-42 = 53% of fluorescence) and **4b** (Aβ1‐42 = 50% and AβpE3‐42 = 90% of fluorescence) from the first publication and compound **4c** (Aβ1‐42 = 74% and AβpE3‐42 = 51% of fluorescence) from the second one (Figure 6). The latter was able to slightly improve activity towards AβpE3-42 but accomplishing increased solubility.

Due to the multifactorial pathogenesis of AD, one of the current drug discovery approaches is the so-called multitarget-directed ligands (MTDLs). In 2015, Benchekroun et al. [38] reported the design, synthesis, and biological evaluation of a new family of ferulic acid–tacrine hybrids (FATHs), using the Ugi reaction. FATHs were selected because tacrine is a well-known cholinesterase (ChE) inhibitor, although it is hepatotoxic, and ferulic acid is a potent antioxidant.

**Scheme 5:**
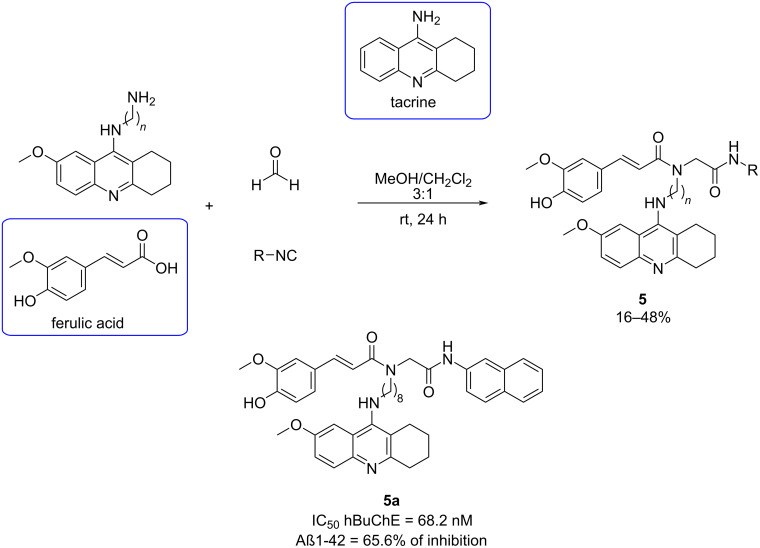
Four-component Ugi reaction to obtain FATH hybrids and the best candidate synthesized.

Fourteen FATH were synthesized and tested for hepatotoxicity, cholinesterase inhibition (human BuChE, hBuChE), Aβ1-42 self-aggregation inhibition, and antioxidant activity. FATH **5a** (Scheme 5) emerged as a promising candidate, showing lower hepatotoxicity at 1000 µM (59.4%, 1.72-fold less toxic than tacrine), moderate and selective hBuChE inhibition (IC_50_ = 68.2 nM), strong antioxidant properties and notable inhibition of Aβ1-42 self-aggregation (65.6% of inhibition). These findings suggest FATH **5a** as a potential lead compound for further investigation in AD therapy.

In 2016, the same group developed ferulic acid–tacrine–melatonin hybrids (FATMHs) and lipoic acid–tacrine–melatonin hybrids (LATMHs), deciding to incorporate melatonin as a new target for the MTDL [39]. Melatonin has the ability to combat OS, which is a crucial factor in the pathogenesis of AD. Moreover, it plays a neuroprotective role against Aβ-peptides and easily crosses the blood–brain barrier (BBB). Finally, recent research has implicated melatonin in modulating the Nrf2/ARE pathway, a crucial defense mechanism against OS and inflammation.

**Scheme 6:**
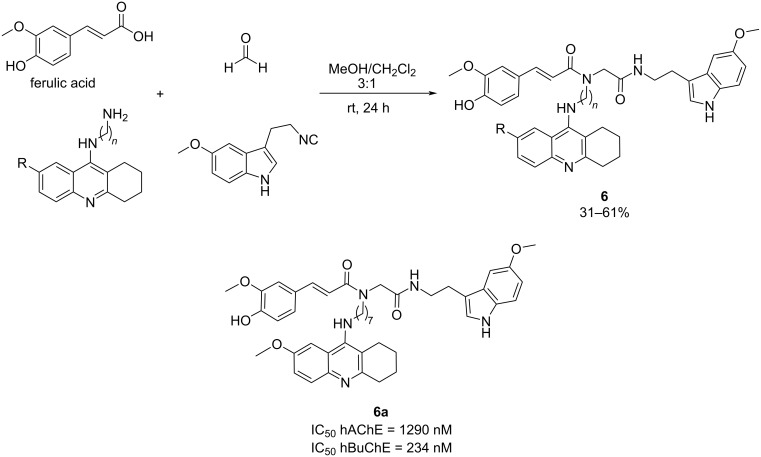
Four-component Ugi reaction for the synthesis of FATMH hybrids and the best candidate synthesized.

In this study, FATMHs and LATMHs were synthesized through Ugi reaction and evaluated for their therapeutic potential in AD. Among these compounds, FATMH **6a** emerged as a remarkable candidate, demonstrating strong antioxidant properties (9.11 TE), potent cholinesterases inhibition, particularly BuChE [IC_50_ (hAChE) = 1290 nM; IC_50_ (hBuChE) = 234 nM], positive non-hepatotoxic profile (at 100, 300, and 1000 μM) compared to tacrine, and favorable neuroprotective effects against toxic insults such as H_2_O_2_ and Aβ-peptides (Scheme 6). Additionally, compound **6a** showed promising activity in activating the Nrf2 signaling pathway, indicating its potential to mitigate OS and inflammation in AD. These findings highlight FATMH **6a** as a promising lead compound for further development as a therapeutic agent for AD.

**Scheme 7:**
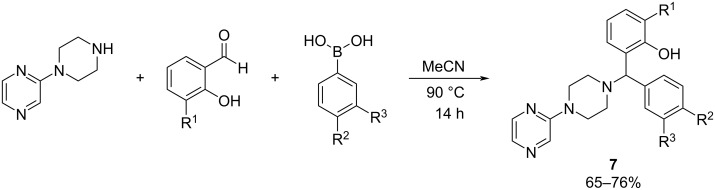
Petasis multicomponent reaction to produce pyrazine-based MTDLs.

**Petasis reaction:** Continuing in the context of the development of MTDLs, in 2023 Madhav et al. [40] reported the synthesis of pyrazine-based MTDLs using the Petasis reaction, which involves a secondary amine, a suitable aldehyde, and a substituted boronic acid in the presence of MeCN, under inert atmospheric conditions at 90 ºC for 14 h (Scheme 7).

**Figure 7:**
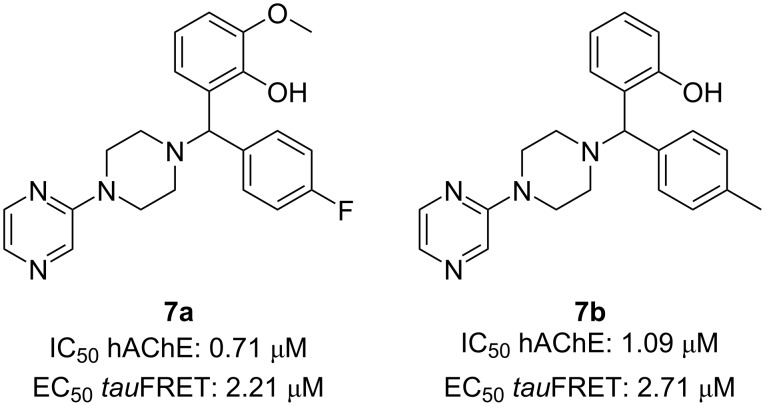
Best pyrazine-based MTDLs synthesized by Madhav et al. [40].

The efficacy of these MTDLs was assessed in terms of their ability to inhibit AChE and tau aggregation, along with their neuroprotective effects on the SH-SY5Y cell line. The evaluation of hAChE inhibitory action was conducted at a concentration of 10 μM, with donepezil serving as a positive control. To measure their impact on cellular tau oligomerization, the fluorescence resonance energy transfer (FRET) signal intensity was quantified in a cellular tau FRET assay at the same 10 μM concentration, using MK-886, a known tau-oligomerization inhibitor, as the positive control. Two compounds (**7a** and **7b**, Figure 7) demonstrated a high inhibitory activity against AChE and tau-oligomerization, which were selected for further exploration.

The neuroprotective effects of the compounds were assessed in comparison to MK-886 and donepezil, employing a widely accepted tauopathy model in SH-SY5Y cells. Notably, compound **7a** was identified as the most promising multitarget-directed ligand in the study, exhibiting superior neuroprotection compared to both MK-886 and the commercial drug donepezil in a cell viability MTT assay. These findings reinforced the theory that developing MTDLs could provide a more effective approach for addressing complex neurodegenerative disorders than conventional single-target strategies.

**Knoevenagel-based multicomponent reactions:** Several quinazolinone derivatives have already been developed as MTDLs, inspired by natural alkaloids such as deoxyvasicinone and evodiamine with promising inhibition of ChEs and antioxidant and neuroprotective effects against glutamate-induced OS in HT-22 cells [41].

**Scheme 8:**
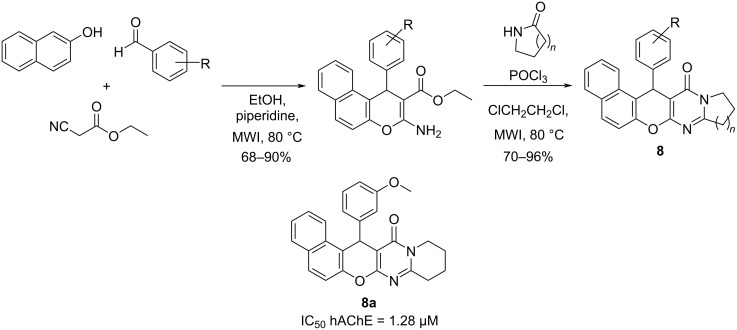
Synthesis of BCPOs employing a Knoevenagel-based multicomponent reaction and the best candidate synthesized.

In 2016, Dgachi et al. [42] applied a Knoevenagel-based multicomponent approach to further explore the quinazolinone scaffold. They have obtained new benzochromenopyrimidinones, abbreviated as BCPOs, whose synthesis has been accomplished in two steps. First, a microwave-assisted reaction of ethyl cyanoacetate, selected aromatic aldehydes, and 2-naphthol was performed. The second step was the condensation of the obtained intermediates with the appropriate commercial lactams, under microwave irradiation (Scheme 8).

As a result, compound **8a** has been identified as a promising derivative potentially useful in further AD drug discovery for its antioxidant activity (4.7 TE in ORAC-FL assay), moderate hAChE inhibition (IC_50_ = 1.28 μM), and non-toxicity in liver HepG2 cells.

**Scheme 9:**
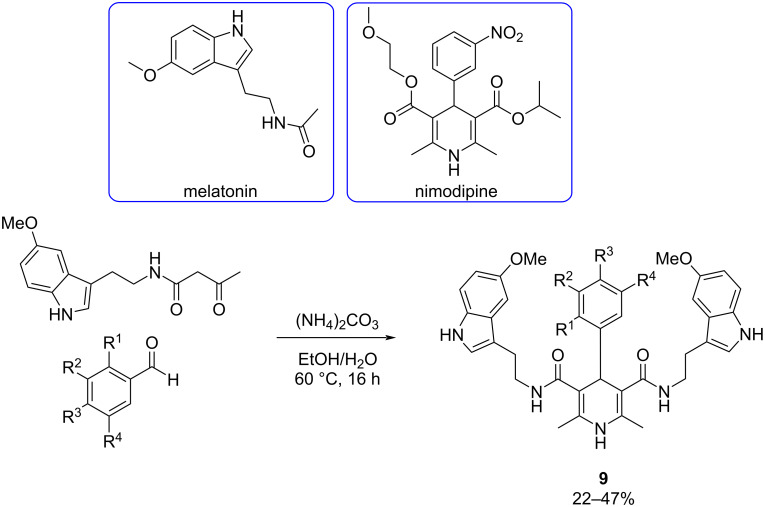
Hantzsch multicomponent reaction for the synthesis of DHPs as novel MTDLs.

**Hantzsch-based strategies:** As mentioned before, in response to the challenges posed by the multifactorial nature of AD, the MTDL approach has emerged as a promising strategy. This approach involves designing drugs capable of binding simultaneously to various enzymatic systems or receptors implicated in AD pathology. In 2019, Malek et al. [43] designed and obtained a new family of 1,4-dihydropyridines (DHPs), as a series of MTDLs, which were prepared using a multicomponent reaction, particularly the Hantzsch reaction (Scheme 9).

The synthesized DHPs result from the juxtaposition of nimodipine, a well-known calcium channel blocker and melatonin, a reputed antioxidant agent. This approach lies in the known association between increased cytosolic calcium levels and the formation of Aβ peptides, which are implicated in AD pathology. Calcium also regulates kinases involved in the hyperphosphorylation of tau, contributing to neurofibrillary tangles. Meanwhile, melatonin is known for its neuroprotective properties, countering oxidative stress by scavenging radical oxygenated species and exhibiting potent antioxidant capacity [44–45].

The calcium channel blockade was determined by the measure of Ca^2+^ influx induced by K^+^-depolarization in SH-SY5Y neuroblastoma cells, previously loaded with the fluorescent dye Fluo-4AM. On the other hand, the antioxidant activity of the DHPs was evaluated using the ORAC-FL method.

**Figure 8:**
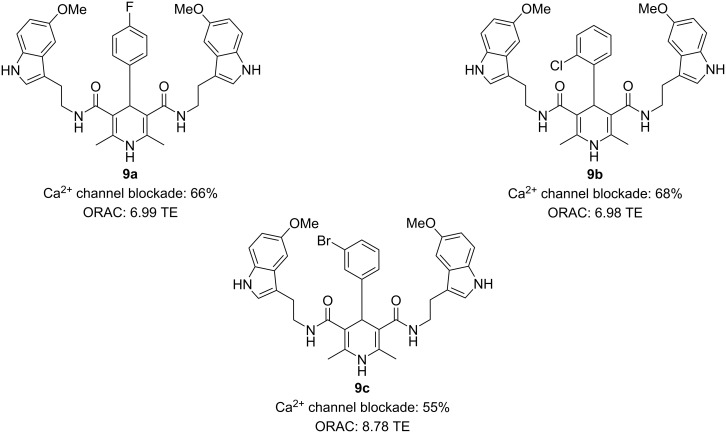
Most active 1,4-dihydropyridines developed by Malek et al. [43].

The most potent compounds, particularly DHPs **9a**, **9b**, and **9c**, demonstrated significant efficacy (Figure 8). DHP **9c** emerged as the most balanced MTDLs, being the most potent antioxidant and showing equipotency with nimodipine as a calcium channel blocker, which suggests that DHP **9c** may be promising for potential AD treatment.

**Scheme 10:**
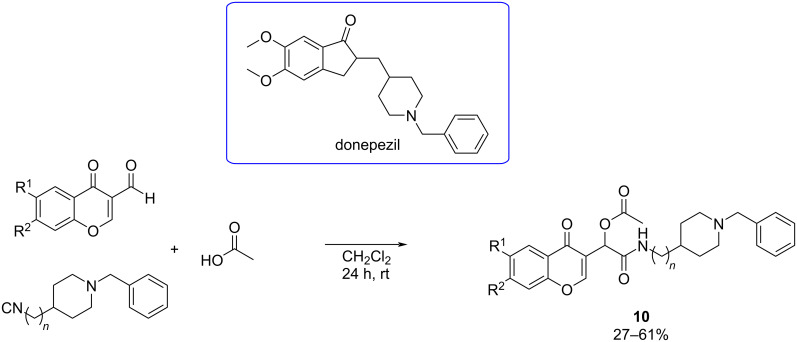
Chromone–donepezil hybrid MTDLs obtained via the Passerini reaction.

**Passerini reaction:** Based on the power of multicomponent reactions to afford rapidly and efficiently chemical diversity and continuing with an effort to develop new MTDL, Malek et al. [46] described in 2019 the design, synthesis, and the biological evaluation of new racemic chromone donepezil hybrids (CDHs) via Passerini reaction (Scheme 10), combining the antioxidant properties of chromone with the cholinesterase inhibitor (ChEI) donepezil.

**Figure 9:**
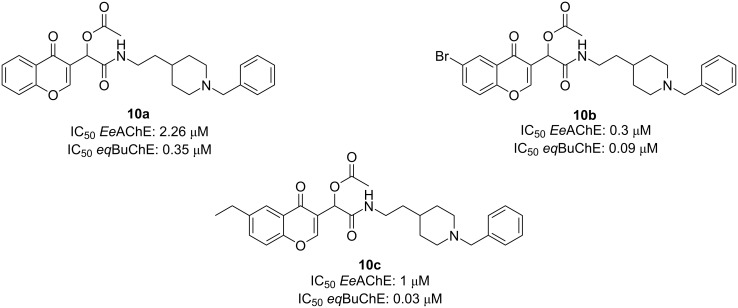
Best CDH-based MTDLs as AChE inhibitors synthesized by Malek et al. [46].

The antioxidant activity of the synthesized compounds was evaluated using the ORAC-FL method and the anticholinesterase activity against EeAChE and eqBuChE using Ellman’s protocol (Figure 9). CDH **10b** emerged as a potent EeAChEI (IC_50_ = 0.30 μM) and CDH **10c** as a strong eqBuChEI (IC_50_ = 0.03 μM), both showing promising activity compared to tacrine and donepezil. A SAR analysis indicated the linker length's crucial role in CDH inhibition activity, as all the compounds with *n* = 2 were more potent. All compounds were very modest antioxidants compared to ferulic acid, showing values ranging between 0.42–1.27 TE with derivative **10a** being the most potent antioxidant.

The amyloid hypothesis, which states that β-amyloid (Aβ) aggregates cause the onset and progression of AD, is a leading proposal to explain AD etiology. Based on this hypothesis, compounds that inhibit γ-secretase, one of the enzymes responsible for forming Aβ, are potential therapeutics for AD.

**Scheme 11:**
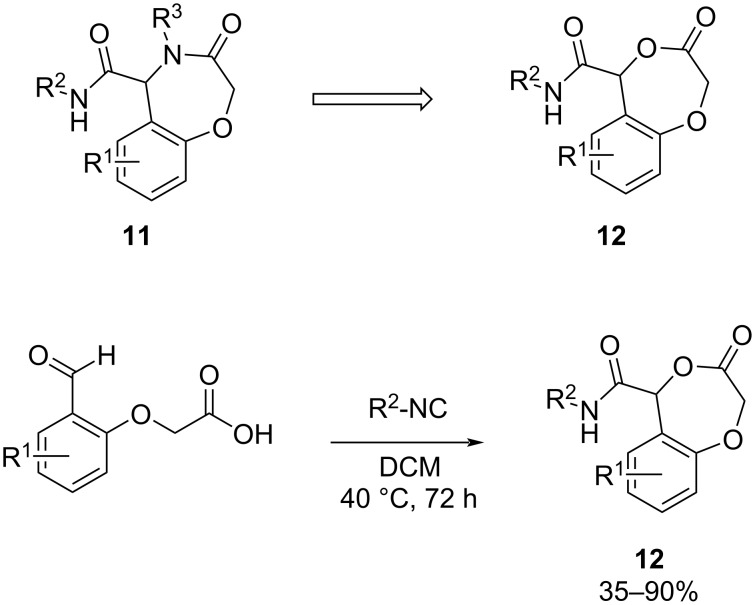
Replacement of the nitrogen in lactams **11** with an oxygen in **12** to influence hydrogen-bond donating properties and synthesis of the benzodioxepinone derivatives via Passerini reaction.

In 2024, Fragkiadakis et al. [47] developed a rapid synthesis of benzodioxepinones. The attention was focused on the oxazepane scaffold **11** (Scheme 11). Replacing the nitrogen in the bioactive lactam **11** (γ-secretase inhibitor in AD therapy) with an oxygen should reduce the hydrogen bond donating (HBD) ability of the compounds.

The method employs readily available starting materials, resulting in the desired compounds in a single step. The oxazepine scaffold can be easily accessed using the Ugi four-component reaction. By modifying this scaffold, the researchers aimed to reduce the hydrogen-bond donating properties. The Passerini reaction, employing bifunctional salicylaldehydes and isocyanides successfully yielded benzodioxepinones **12** in all cases (Scheme 11). The synthesized compounds demonstrated excellent drug-like features.

#### Parkinson disease (PD)

**Scheme 12:**
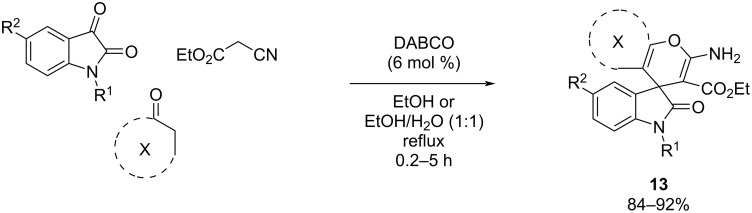
MCR 3 + 2 reaction to develop spirooxindole, spiroacenaphthylene, and bisbenzo[*b*]pyran compounds.

**Knoevenagel–Michael addition/cyclization (MCR 3 + 2):** Sirtuins, a group of enzymes that rely on NAD^+^ to function as protein deacetylases, have garnered attention across multiple areas, including lifespan extension, obesity, age-associated diseases, neurological function, cardiovascular disorders, and cancer. Notably, the inhibition of sirtuin 2 (SIRT2) has demonstrated protective effects in both primary neuronal cultures and invertebrate models of PD [48]. SIRT2 is present in various tissues, with a prominent expression in the adult brain, where it contributes to metabolic processes, aging, and epigenetic regulation. In this context, the group of Hasaninejad et al. (2017) [49] described the synthesis of spirooxindole, spiroacenaphthylene, and bisbenzo[*b*]pyran derivatives using a multicomponent reaction to obtain SIRT2 inhibitors (Scheme 12). The authors suggest a Knoevenagel condensation approach between isatin derivatives and ethyl cyanoacetate, followed by a Michael addition with C–H activated carbonyl compounds and intramolecular cyclization [50]. They synthesized 45 compounds with high yields (84–92%). The three most promising compounds of the library (**13a**, **13b**, and **13c**) were selected for further detailed characterization. In vitro evaluation was performed employing a high-performance liquid chromatography (HPLC)-based methodology, using the fluorogenic histone deacetylase substrate MAL to determine the inhibitory activity, and using sirtinol as positive control.

**Figure 10:**
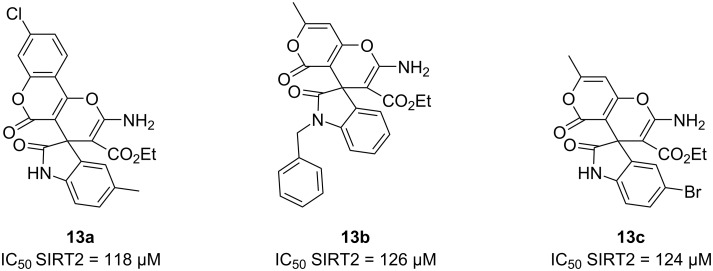
SIRT2 activity of best derivatives obtained by Hasaninejad et al. [49].

When compared to sirtinol (IC_50_ = 67 µM), selected compounds showed moderate affinity towards SIRT2, with IC_50_ values ranging from 118 to 126 µM (Figure 10).

**Gewald reaction:** Cannabinoid receptors have been proposed as promising neuroprotective targets in several chronic progressive disorders such as AD and PD [51]. Recently, in 2023, Figuerola-Asencio et al. [52] synthesized a series of novel compounds derived from ML192, an antagonist ligand for GRP55. This receptor responds to certain cannabinoids, suggesting it can be part of the endocannabinoid system. GRP55 is extensively distributed throughout the CNS, notably in regions such as peripheral tissues, hippocampus, thalamus, and cerebellum, and in the basal ganglia. The therapeutic potential of GRP55 receptor modulators makes it necessary to search for new selective non-cannabinoid ligands due to the important motor impairment found in mice lacking GPR55 [51].

**Scheme 13:**
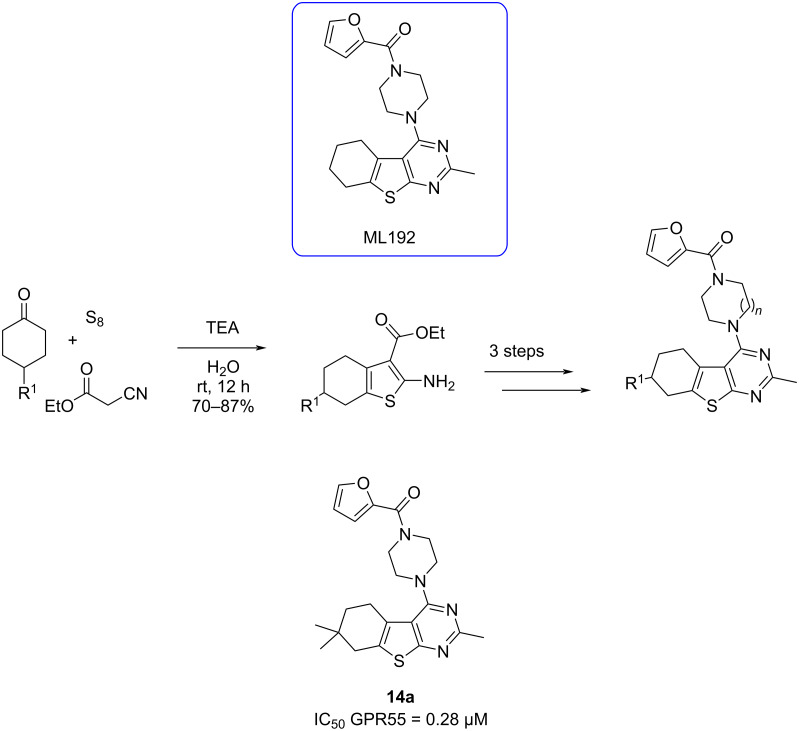
Synthesis of ML192 analogs using the Gewald multicomponent reaction and the best candidate synthesized.

In this context, ML192 analogs were synthesized employing a divergent synthetic route starting with a multicomponent reaction, the Gewald reaction (Scheme 13). This reaction allows to easily build the principal heterocycle of the compounds. Good reaction yields (70–87%) made it possible to obtain the aminothiophene carboxylates necessary for the following steps of the route [52].

Some of the intermediates of the Gewald reaction were oxidized to provide further exploration of the heterocyclic core. The novel compounds were first evaluated using β-arrestin recruitment assays in CHO (chinese hamster ovary) cells overexpressing human GPR55. These new compounds have been evaluated in competitive binding assays for cannabinoid receptors, but all of them showed to be selective for GPR55 (>4 μM for cannabinoid receptors). Then, the most promising antagonist (**14a**; IC_50_ = 0.28 µM) was selected for a G-protein-dependent functional assay, where **14a** acted as a weak GPR55 inverse agonist (EC_50_ = 0.9 µM).

#### Depression and anxiety

Benzodiazepines are widely used in treating a range of CNS disorders, including epilepsy, muscle relaxation, insomnia, anesthesia, anxiety, and depression [53]. The common coexistence of depression and anxiety may indicate the simultaneous presence of mood and anxiety disorders. Clinical observations imply that benzodiazepines may have utility in treating depression, particularly when accompanied by anxiety. In the initial four weeks of treatment, combining medications proved more effective in reducing depressive symptoms and achieving response and remission of major depression, while showing comparable results in reducing anxiety [54].

Recent advancements in organic synthesis, particularly through MCRs, have streamlined the synthesis of benzodiazepines, making it more efficient and environmentally friendly [55]. For example, olanzapine as a benzodiazepine-based clinical drug was appropriately and concisely synthesized via MCR [56–57].

Over the last twenty years, the Ugi reaction has emerged as a highly considered reaction due to its mild conditions, broad applications, and product diversity. It enables the selective assembly of precursors, facilitating various post-reaction transformations such as deprotection cyclization, 1,3-dipolar cycloaddition, and intramolecular SNAr reactions.

**Ugi-4CR/deprotection/cyclization (UDC) strategy:** In the UDC approach a protected amine and an electrophilic functional group like esters or ketones are used. Convertible isocyanides can also be employed. Once the Ugi reaction is completed, the protecting group is removed, and the heterocyclic scaffold is subsequently formed [58].

**Scheme 14:**
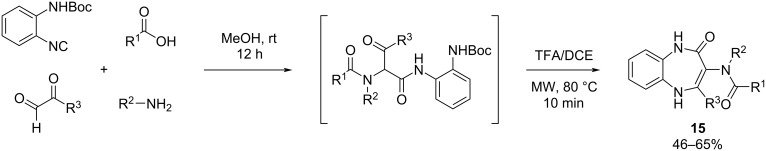
Development of 1,5-benzodiazepines via Ugi/deprotection/cyclization (UDC) approach by Xu et al. [59].

In 2015, Xu et al. [59] reported the synthesis of 1,5-benzodiazepines using the UDC approach. The process begins with Ugi-4CRs, incorporating amines, glyoxaldehydes, 2-(*N*-Boc-amino)phenylisocyanide, and carboxylic acids. Following this, the Boc group is eliminated through microwave-assisted treatment under acidic conditions, resulting in the intramolecular cyclization of the amine onto the ketone derived from glyoxaldehyde (Scheme 14).

**Scheme 15:**
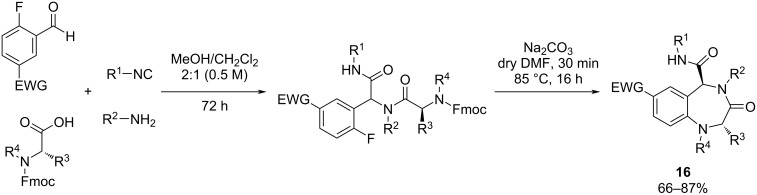
Synthesis of polysubstituted 1,4-benzodiazepin-3-ones using UDC strategy.

Vézina-Dawod and co-workers [60] introduced a strategy, which consists of two reactions, for synthesizing highly substituted 1,4-benzodiazepin-3-ones using the UDC method. This process involves *N*-Fmoc-amino acids, isocyanides, amines, and derivatives of 2-fluorobenzaldehyde (Scheme 15).

**Ugi-4CR/reduction/cyclization (URC) strategy:** The URC pathway enables the synthesis of benzodiazepines by using amine surrogates, such as nitro or azide groups, rather than protected amines. Following the Ugi-4CR, the nitro or azide group is reduced to form the amine, leading to a condensation reaction that results in the formation of the benzodiazepine ring [55].

**Scheme 16:**
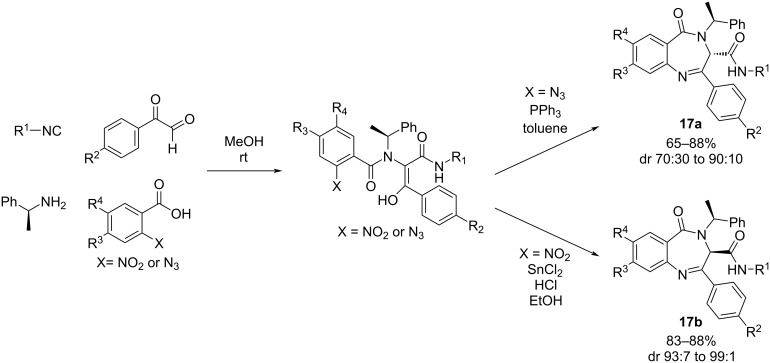
Synthetic procedure to obtain 3-carboxamide-1,4-benzodiazepin-5-ones employing Ugi–reduction–cyclization (URC) approach.

Pertejo et al. [61] described the diastereselective synthesis of 3-carboxamide-1,4-benzodiazepin-5-ones when enantiopure (*S*)-(−)-α-methylbenzylamine and arylglyoxals were used. Thus, a reversal of diastereoselectivity was observed depending on the cyclization methodology employed, the reduction of a nitro group or the Staudinger/aza-Wittig on azide derivatives. This strategic approach allows precise control of the stereogenic centers at the C3-position during cyclization (Scheme 16).

**Scheme 17:**
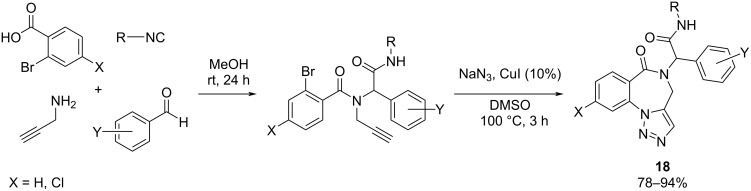
Ugi cross-coupling (U-4CRs) to synthesize triazolobenzodiazepines.

**Ugi-4CR/metal-catalyzed reaction:** The fusion of MCRs with cross-coupling reactions represents an attractive approach as a result of the increased intricacy and effectiveness [62]. Moreover, this strategy may offer a broad selection of compounds with biological interest. Asgari et al. [63] described an efficient two-step approach for the synthesis of triazolobenzodiazepines in good to excellent yields (Scheme 17). The protocol is initiated by Ugi-4CRs followed by sequential click/intramolecular Ullmann-type C–N coupling reactions.

**Scheme 18:**
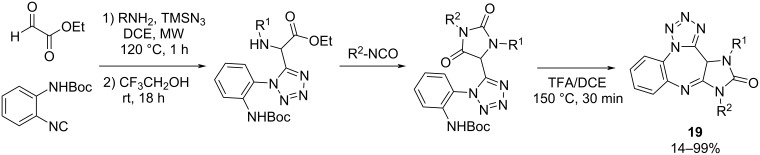
Azido-Ugi four component reaction cyclization to obtain imidazotetrazolodiazepinones.

**Azido-Ugi 4CR/cyclization (AU-4CR) strategy:** The azido-Ugi four-component reaction (AU-4CR) is an elegant and atom economical approach for obtaining substituted tetrazoles, which are very relevant in medicinal chemistry [64]. When combined with suitable post-Ugi transformations, it is possible to obtain diverse heterocyclic compounds with tetrazole moieties [65]. In this context, Medda et al. [66] developed a three-step process to access biologically active imidazotetrazolodiazepinones. The first one is the AU-4CRs among ethyl glyoxalate, amines, isocyanide, and TMSN_3_, after which the Ugi adduct is treated with an excess of different isocyanates resulting in the corresponding hydantoin derivatives. Finally, under microwave irradiation, the Boc group is removed under acid conditions and the benzodiazepine core is formed after ring closure (Scheme 18).

**Scheme 19:**
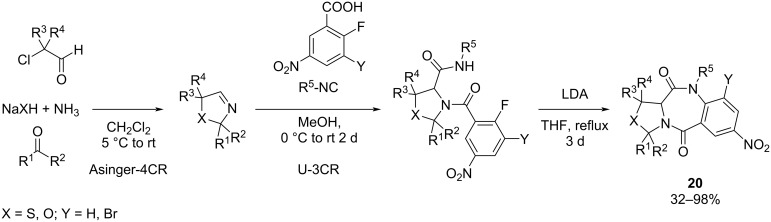
Synthesis of oxazolo- and thiazolo[1,4]benzodiazepine-2,5-diones via Ugi/deprotection/cyclization approach.

**Ugi-3CR/deprotection/cyclization strategy:** Kröger and colleagues [67] reported a novel synthetic pathway for synthesizing a series of thiazolo- and oxazolo[1,4]benzodiazepine-2,5-diones employing combinatorial MCRs. The first step is the Asinger-4CR, which involves a ketone, α-chloroaldehyde, ammonia, and either sodium hydroxide or sodium hydrogen sulfide to obtain a cyclic imine. Subsequently, the U-3CR is performed, where the cyclic imine reacts with an electron-deficient 2-fluorobenzoic acid and an isocyanide to yield a bisamide. Then, the bisamide undergoes an intramolecular SNAr reaction to form the oxazolo- and thiazolo-fused benzodiazepinediones (Scheme 19).

#### Schizophrenia

As previously mentioned in the introduction, the pathophysiology of schizophrenia remains unclear, and therefore, there are numerous hypotheses to explain it, including the dopaminergic and glutamatergic pathways, among others.

**Ugi reaction:** The D_2_ dopamine receptor (DRD2) is the target of different drugs, including antipsychotics, antiparkinsonian agents, and addiction-related disorders. Aripiprazole and cariprazine are prototypes of newer antipsychotic drugs with a bitopic ligand structure, which includes both primary and secondary pharmacophores. Inspired by the structure and bioactivity profile of aripiprazole, a novel series of DRD2-biased agonists have been developed. The study involves the design, synthesis, and pharmacological characterization of DRD2 partial agonists [68].

**Scheme 20:**
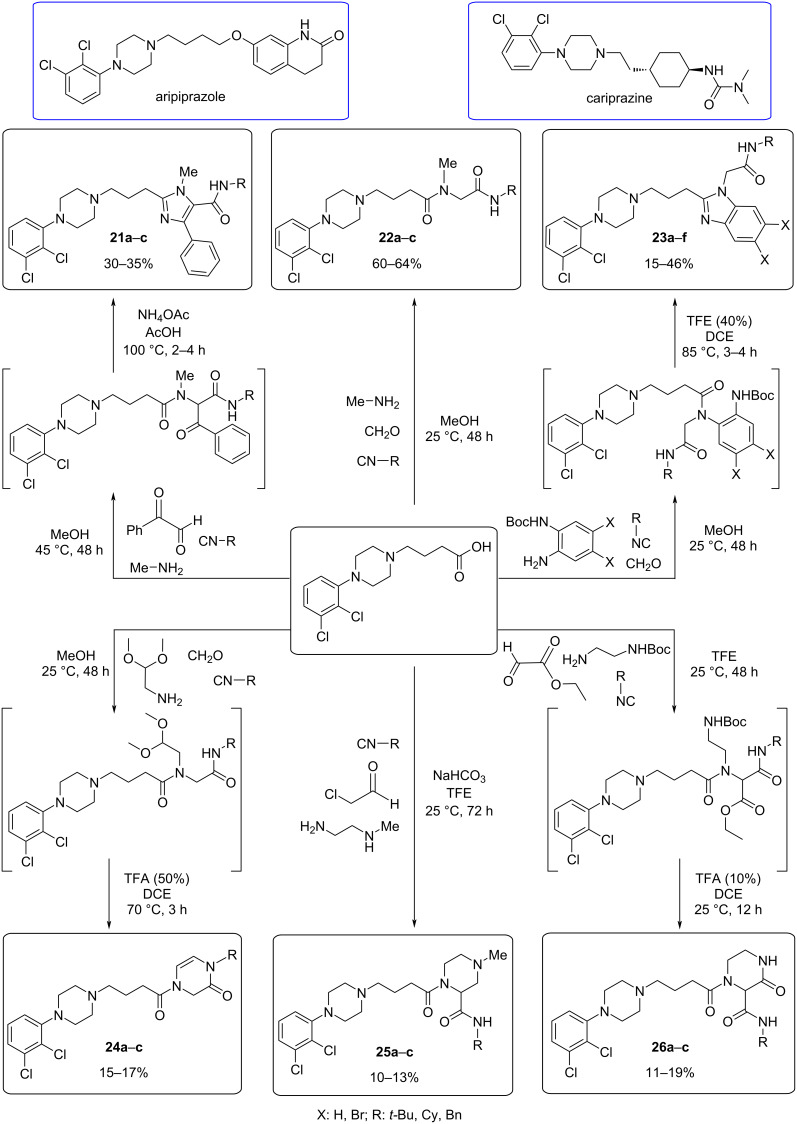
General synthesis of 2,3-dichlorophenylpiperazine-derived compounds by the Ugi reaction and Ugi/deprotection/cyclization approach.

**Figure 11:**
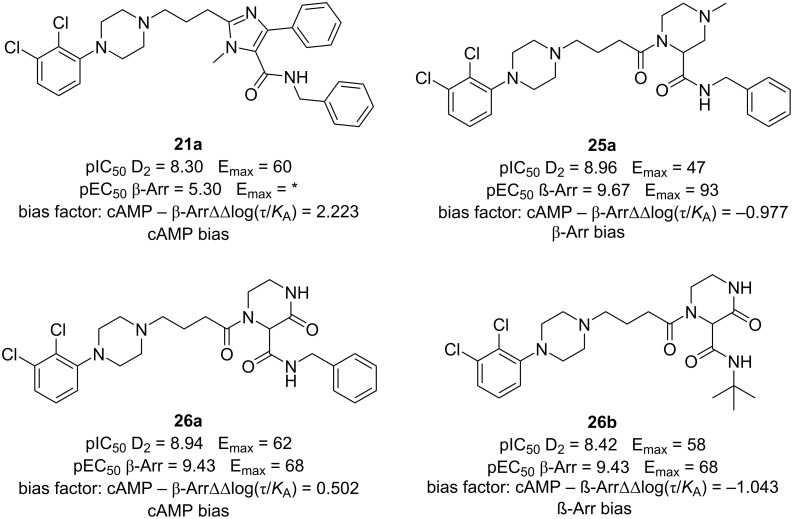
Best DRD2 compounds synthesized using a multicomponent strategy.

Employing Ugi and Ugi post-cyclization reactions, 21 novel 2,3-dichlorophenylpiperazine-derived compounds were synthesized (Scheme 20). These novel compounds were evaluated for their activity on the DRD2 receptor through cAMP functional assays. Furthermore, the most promising ligands were selected for further investigation using β-arrestin-2 recruitment assays. Ligands **21a** and **26a** showed a significant bias towards the cAMP pathway, while ligands **25a** and **26b** exhibited β-arrestin biased agonism (Figure 11). These findings demonstrate the potential for developing functionally selective DRD2 ligands with diverse signaling profiles using a multicomponent approach.

**Bucherer–Bergs reaction:** The dense presence of mGlu2/3 receptors in regions associated with psychiatric disorders suggests them as potential drug targets. One clinical candidate to treat psychiatric disorders was the mGluR2/3 agonist pomaglumetad methionil (POM). This drug was withdrawn from phase III clinical trials due to insufficient efficacy compared to current antipsychotic drugs (APDs). However, POM demonstrated to be effective to treat certain populations [69].

**Scheme 21:**
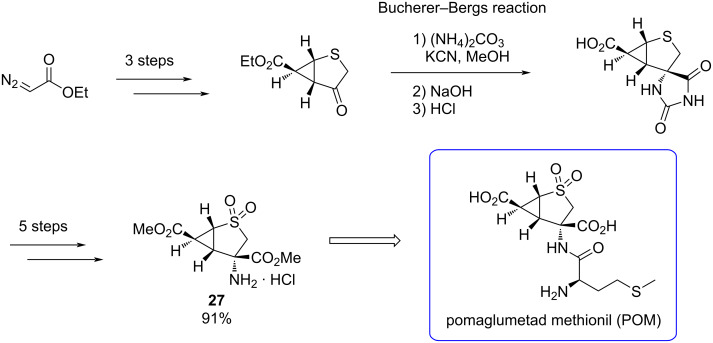
Bucherer–Bergs multicomponent reaction to obtain a key intermediate in the synthesis of pomaglumetad methionil (POM).

The large-scale synthesis of a key intermediate of POM was described by Waser et al. [70] in 2011. In this study, they employed the Bucherer–Bergs reaction, a versatile multicomponent reaction which enables the formation of a hydantoin that represents a masked amino acid functionality (Scheme 21). After subsequent saponification and acidification, the desired compound crystallizes along with impurities from other isomers. The undesired isomers were removed in later steps.

In 2020, Sonnenschein et al. [69] studied the effects of POM on dopaminergic neuron activity in states of increased ventral hippocampus (vHPC) activity, measuring the electrical activity of neurons in the brain of rats. Their findings illustrated that POM has the capability to restore normal dopamine neuron activity in situations of increased hippocampal activity. These results showed that a deep understanding of the mechanistic function of mGlu2/3, and particularly the agonist POM, is needed.

#### Epilepsy

Epilepsy is a disease caused by several biological causes, such as uncontrolled inflammation, neurodegeneration, and aberrant neurogenesis. Levetiracetam is the first member of a new class of anti-epileptic drugs, which selectively accumulates rapidly firing neurons and inhibits their activity. This drug binds to SV2A, a synaptic vesicle protein that is required for normal synaptic function, interfering with the release of the neurotransmitter [71–72].

**Scheme 22:**
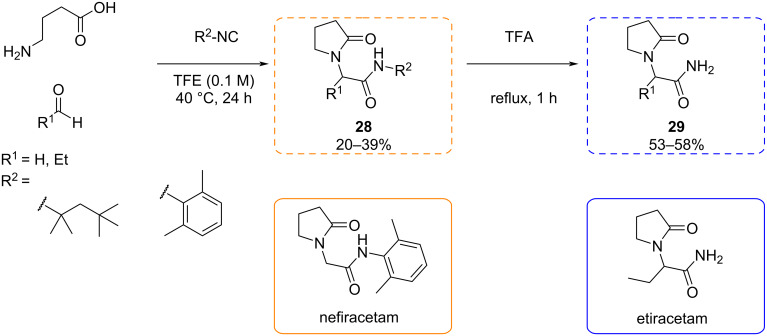
Ugi reaction to synthesize racetam derivatives and example of two racetams synthesized by Cioc et al. [73].

**Ugi reaction:** Cioc et al. [73] recognized the 2-oxo-1-pyrrolidino acetamide structure of levetiracetam as a potential Ugi scaffold and aimed to construct racetam derivatives through a multicomponent condensation. While previous Ugi approaches toward racetam derivatives existed, they typically involved indirect grafting of the pyrrolidone core. In this study, the authors synthesized the 2-oxo-1-pyrrolidino acetamide scaffold directly from γ-aminobutyric acid, a carbonyl compound, and an isocyanide in a single operation. The reaction proved to be effective using different isocyanides, both aromatic and aliphatic ones. Additionally, the transformation worked with the convertible isocyanide. This opened the possibility of synthesizing various derivatives of racetams (Scheme 22). On the other hand, they demonstrated the feasibility of the reaction for the synthesis of different racetams useful in the clinic, such as etiracetam (racemic form of (*S*)-levetiracetam) and nefiracetam, another antiepileptic with racetam structure and similar properties [74].

## Conclusion

This review highlights the remarkable advantages of multicomponent reactions (MCRs) as a powerful tool in the rapid development of pharmacologically active compounds, particularly for addressing critical nervous system disorders. The ability of MCRs to generate diverse chemical libraries in a short time frame makes them invaluable in accelerating drug discovery processes. The synergy between multicomponent chemistry and medicinal chemistry offers a highly attractive and competitive approach to accelerate drug discovery and development, facilitating the identification and the optimization of novel therapeutic agents for central nervous system pathologies. This combination not only enhances the pace of discovery but also broadens the scope of potential treatments, underscoring the significant impact of MCRs in medicinal chemistry.

## Data Availability

Data sharing is not applicable as no new data was generated or analyzed in this study.
